# Association between Relative Bradycardia and Adult-onset Still Disease

**DOI:** 10.31662/jmaj.2023-0189

**Published:** 2024-06-10

**Authors:** Takao Wakabayashi, Hiroyoshi Iwata

**Affiliations:** 1 Department of General and Emergency Medicine, Japan Community Health-Care Organization Sapporo Hokushin Hospital, Sapporo, Japan; 2 Center for Environmental and Health Sciences, Hokkaido University, Sapporo, Japan

**Keywords:** adult-onset Still disease, macrophage, physical examination, relative bradycardia, vital sign

## Introduction

Relative bradycardia is a significant physiological vital sign of critical diseases ^(1)^. The heart rate usually increases by 18 beats/min (bpm) for every degree increase in temperature (degrees Celsius) ^(2)^. In relative bradycardia, the pulse rate does not increase despite a rise in body temperature. Relative bradycardia is observed in infectious diseases, such as Legionella pneumonia, lymphoma, and central nerve fever ^(1)^. Therefore, the presence of relative bradycardia facilitates the diagnosis of these diseases.

Adult-onset Still disease (AOSD) causes fever of unknown origin ^(3)^. The clinical features of AOSD resemble those of various infectious diseases, thus leading to diagnostic difficulties. Furthermore, patients with AOSD present with a multisystemic disorder of indeterminate etiology, causing diagnostic difficulties and limiting scholarly investigations compared with other rheumatic diseases ^(4)^, despite the unfavorable prognosis of patients with severe AOSD.

To the best of our knowledge, only a few studies have determined whether a patient with AOSD potentially exhibits relative bradycardia. Thus, we aimed to determine the association between AOSD and relative bradycardia. If relative bradycardia occurs in patients with AOSD, this vital sign may facilitate the diagnosis of AOSD.

## Methods

### Patients and analysis

Between April 1, 2016, and March 31, 2023, we enrolled all eligible inpatients aged ≥16 years with newly diagnosed AOSD in an acute care hospital. We retrospectively obtained the patients’ baseline characteristics, including age, sex, underlying disease, Yamaguchi criteria, hospitalization, vital sign, relative bradycardia, clinical manifestation, and laboratory data, from clinical charts for each case. In addition, we collected the pulse rate and body temperature of patients who met the following criteria: 1) within the first 5 days after admission, 2) without therapeutic intervention, and 3) simultaneous measurement. We defined Δ pulse rate as pulse rate minus 110 per minute and Δ body temperature as body temperature minus 38.3°C. Moreover, we created scatter plots and conducted linear regression analysis with coefficient determination (R^2^). The predicted line according to Cunha’s criteria was added to the figure.

### Diagnostic criteria and exclusion criteria

AOSD was diagnosed based on the Yamaguchi criteria ^(5)^. The Yamaguchi criteria consist of four major items (1) temperature >39°C lasting 1 week, 2) arthralgia or arthritis lasting 2 weeks, 3) typical rash, and 4) leukocytosis >10,000 with 80% polymorphonuclear cells) and four minor items (1) sore throat, 2) lymphadenopathy or splenomegaly, 3) abnormal liver function test, and 4) negative antinuclear antibody and rheumatoid factor tests). At least five items should be fulfilled to diagnose AOSD, including a minimum of two major items.

Relative bradycardia was defined using Cunha’s criteria ^(1)^ as follows: 1) body temperature >38.3°C and 2) pulse below the upper pulse limit for the body temperature (38.3°C for 110 bpm, 39.4°C for 120 bpm, 40.0°C for 130 bpm, 40.6°C for 140 bpm, and 41.1°C for 150 bpm). If the body temperature fell between these temperatures, relative bradycardia was determined by the pulse rate for the next lowest temperature (e.g., 38.7°C, rounded down to 38.3°C for 110 bpm). Body temperatures were evaluated at the axilla ^(6)^.

### Ethics

This study was approved by the Institutional Review Board of the surveyed hospital (IRB 2021-9). Informed consent was obtained in the form of opt-out on the website.

The exclusion criteria were as follows: treatment with heart rate-altering agents (β-blockers, nondihydropyridine calcium channel blockers, antiarrhythmic drugs, or cilostazol), steroid therapy before hospitalization, conditions associated with bradycardia (e.g., hypokalemia < 3.0 mmol/L), a history of atrial fibrillation, and pacemaker implantation.

## Results

**Figure 1.**
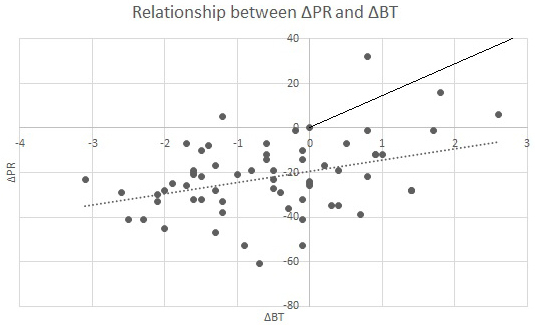
Relationship between ΔPR and ΔBT The dashed line represents the approximation line, whereas the solid line represents the line predicted by Cunha’s criteria. ΔPR, pulse rate minus 110 per minute; ΔBT, body temperature minus 38.3°C.

During the study period, nine patients were diagnosed with AOSD and were enrolled in the study. One patient failed to meet the inclusion criteria; thus, eight patients were included in the final analysis. The patients’ baseline characteristics and the Yamaguchi criteria are shown in Table 1. The mean age of the patients was 59.9 years. The patient cohort included three men with an average age of 38.0 years and five women with an average age of 73.0 years. The underlying diseases were diverse. All patients fulfilled the Yamaguchi criteria. The mean number of days from onset to hospitalization was 9.25 days, and the mean number of days from onset to diagnosis was 13.4 days (Table 2). Five patients (62.5%) met the criteria for relative bradycardia on admission, and all patients met the criteria after day 2 of hospitalization. In all patients, spiking fever was confirmed and serum ferritin levels were above normal (normal range, 16-160 ng/mL), with a mean value of 16,332 ng/mL. Figure 1 presents a scatter plot of the Δbody temperature and Δpulse rate with the approximation line and the predicted line by the Cunha criteria. The regression equation was ΔPR = −19.49 + 5.08 × ΔBT (R^2^ = 0.14).

**Table 1.** Baseline Characteristics and the Yamaguchi Criteria.

		**Overall (N = 8)**
**Baseline characteristics**	
Age			
	Mean [Min, Q1, Median, Q3, Max]	59.9 [30.0, 43.0, 65.5, 71.0, 90.0]
Sex			
	Female	5 (62.5%)
	Male	3 (37.5%)
Underlying disease			
Cataract, Glaucoma		1 (12.5%)
Depression, Hypertension, Hyperlipidemia, Uterine cervix cancer		1 (12.5%)
Femoral neck fracture		1 (12.5%)
Knee prosthesis, DM, Hypertension		1 (12.5%)
Rheumatoid arthritis, Hyperlipidemia		1 (12.5%)
Sinusitis		1 (12.5%)
None		2 (25.0%)
**Yamaguchi criteria**			
Major-1: Temperature >39℃ 1w			
	Yes	8 (100%)
	No	0 (0%)
Major-2: Arthralgia or arthritis 2w			
	Yes	7 (87.5%)
	No	1 (12.5%)
Major-3: Typical rash			
	Yes	4 (50.0%)
	No	4 (50.0%)
Major-4: Leukocytosis >10000 with 80% polymorphonuclear cells			
	Yes	6 (75.0%)
	No	2 (25.0%)
Minor-1: Sore throat			
	Yes	7 (87.5%)
	No	1 (12.5%)
Minor-2: Lymphadenopathy or splenomegaly			
	Yes	1 (12.5%)
	No	7 (87.5%)
Minor-3: Abnormal liver function test			
	Yes	8 (100%)
	No	0 (0%)
Minor-4: Negative tests for ANA and RF			
	Yes	7 (87.5%)
	No	1 (12.5%)

Q1: 25 percentile, Q3: 75 percentile, ANA: antinuclear antibody, RF: rheumatoid factor, DM: diabetes mellitus The Yamaguchi criteria include 4 major and 4 minor criteria. For the diagnosis of adult-onset Still disease, at least 5 criteria must be met, including at least 2 major criteria.

**Table 2.** Clinical Manifestation and Laboratory Data.

	**Overall participants (N = 8)**
**Hospitalization**	
Number of days from onset to hospitalization	
Mean [Min, Q1, Median, Q3, Max]	9.25 [2.0, 2.5, 11.5, 14.0, 15.0]
Number of days from onset to diagnosis	
Mean [Min, Q1, Median, Q3, Max]	13.4 [3.0, 7.25, 15.0, 18.5, 21.0]
**Vital sign and relative bradycardia**	
Body temperature on admission	
Mean [Min, Q1, Median, Q3, Max]	38.3 [36.6, 37.8, 38.5, 39.2, 39.2]
Pulse rate on admission	
Mean [Min, Q1, Median, Q3, Max]	93.1 [75.0, 84.5, 93.0, 104, 110]
Relative bradycardia on admission	
Yes	5 (62.5%)
No	3 (37.5%)
Relative bradycardia after day 2 of hospitalization	
Yes	8 (100%)
No	0 (0%)
**Clinical manifestation and laboratory data**	
Spiking fever	
Yes	8 (100%)
No	0 (0%)
Rigor	
Yes	1 (12.5%)
No	7 (87.5%)
Headache	
Yes	3 (37.5%)
No	5 (62.5%)
Ferritin (ng/mL)	
Mean [Min, Q1, Median, Q3, Max]	16332 [552, 2355, 4682, 15881, 89430]

Q1: 25 percentile, Q3: 75 percentile

## Discussion

Our preliminary investigation demonstrated the occurrence of relative bradycardia at the onset of AOSD. The patients’ vital signs are presented in Figure 1. In the regression equation, the R^2^ value was classified as moderate. This could be attributed to factors such as the easy variability of pulse rate due to autonomic nervous system excitations like physical activity and psychological stress, the mixing of data without considering age and sex, and other reasons. In other words, relative bradycardia does not occur at any point during the course, and its occurrence needs to be carefully observed. Nevertheless, Figure 1 demonstrates that patients’ pulse rates are lower than the predicted ones. Previously, spiking fever was recognized as a manifestation of AOSD ^(3)^, although the definition of spiking fever has not been established. Contrarily, the criteria for relative bradycardia have been reasonably established, and quantitative evaluation is possible. Thus, the combination of relative bradycardia and conventional diagnostic criteria, such as the Yamaguchi criteria, can enhance the diagnostic precision of AOSD.

Our results raise the question regarding the association of AOSD with relative bradycardia. Ye et al. reported that numerous inflammatory cytokines, such as interleukin (IL)-1, IL-6, and tumor necrosis factor, affect cardiac pacemaker cells, leading to relative bradycardia ^(7)^. The inflammatory cytokines involved in macrophage activation in AOSD and other diseases associated with relative bradycardia, such as Legionella pneumonia and lymphoma, overlap. The occurrence of macrophage activation syndrome has been noted in AOSD ^(4)^. Thus, macrophage activation is potentially involved in the occurrence of relative bradycardia.

This study has several limitations. First, it is known that pulse rate varies with age and sex ^(8)^. Second, more accurate criteria for relative bradycardia need to be developed through prospective research. To demonstrate the utility of relative bradycardia as a physical examination, it is necessary to conduct a prospective study that comprehensively compares diseases that cause fever, such as infectious diseases where relative bradycardia has not been reported, and AOSD via multivariate receiver operating characteristic analysis. However, it is noteworthy that the patient cohort was quite limited. Consequently, future research is warranted to conduct a more comprehensive exploration of the association between AOSD and relative bradycardia.

In conclusion, our findings reveal a positive correlation between AOSD and relative bradycardia in this retrospective study. Our results indicate that relative bradycardia may be useful in the early diagnosis of AOSD. Further prospective studies with a larger group of participants are warranted to investigate the correlation between AOSD and relative bradycardia.

## Article Information

### Conflicts of Interest

None

### Acknowledgement

We thank all members who support our work in Japan Community Health-care Sapporo Hokushin Hospital. The authors would like to thank Enago (www.enago.jp) for the English language review.

### Author Contributions

TW: Data collection, study concept and design, drafting of the manuscript.

HI: Study concept and design, critical review of the manuscript and study supervision.

### Approval by Institutional Review Board (IRB)

This study was approved by the Institutional Review Board of the surveyed hospital; Japan Community Health-care Organization Sapporo Hokushin Hospital (IRB 2021-9). Informed consent was obtained in the form of opt-out on the website.
